# Chemical and protein structural basis for biological crosstalk between PPAR*α* and COX enzymes

**DOI:** 10.1007/s10822-014-9815-2

**Published:** 2014-11-27

**Authors:** Ann E. Cleves, Ajay N. Jain

**Affiliations:** 1 Helen Diller Family Comprehensive Cancer Center, University of California, San Francisco, San Francisco, CA USA; 2 Department of Bioengineering and Therapeutic Sciences, University of California, San Francisco, San Francisco, CA USA

**Keywords:** Off-target prediction, Fibrates, Nuclear hormone receptors, Molecular similarity, Docking, Binding site similarity, Polypharmacology

## Abstract

We have previously validated a probabilistic framework that combined computational approaches for predicting the biological activities of small molecule drugs. Molecule comparison methods included molecular structural similarity metrics and similarity computed from lexical analysis of text in drug package inserts. Here we present an analysis of novel drug/target predictions, focusing on those that were not obvious based on known pharmacological crosstalk. Considering those cases where the predicted target was an enzyme with known 3D structure allowed incorporation of information from molecular docking and protein binding pocket similarity in addition to ligand-based comparisons. Taken together, the combination of orthogonal information sources led to investigation of a surprising predicted relationship between a transcription factor and an enzyme, specifically, PPAR*α* and the cyclooxygenase enzymes. These predictions were confirmed by direct biochemical experiments which validate the approach and show for the first time that PPAR*α* agonists are cyclooxygenase inhibitors.

## Introduction

A number of interesting relationships between drugs and novel targets have been uncovered by ligand-based molecular similarity computations: methadone being revealed as a muscarinic antagonist [1, 2], numerous additional examples of ligand crosstalk among aminergic GPCR receptor subtypes and between those receptors and various transporters of amine-containing ligands [3, 4], and examples that highlight less obvious relationships such as the PARP inhibitor PJ34 specifically inhibiting Pim1 kinase [5]. In this work, we show how protein structural information can be exploited to bolster predictions of polypharmacology from ligand-based computations. The approach combines data from molecular docking, protein binding pocket similarity, 3D structural ligand similarity, and ligand-similarity based on lexical analysis of drug package inserts [6–9]. The combined computational approach identified a clear chemical and structural linkage between perixosome proliferator-activator receptor alpha (PPAR*α*) and the cyclooxygenase (COX) enzymes, which share no sequence homology and have disparate in vivo functions.

**Fig. 1:**
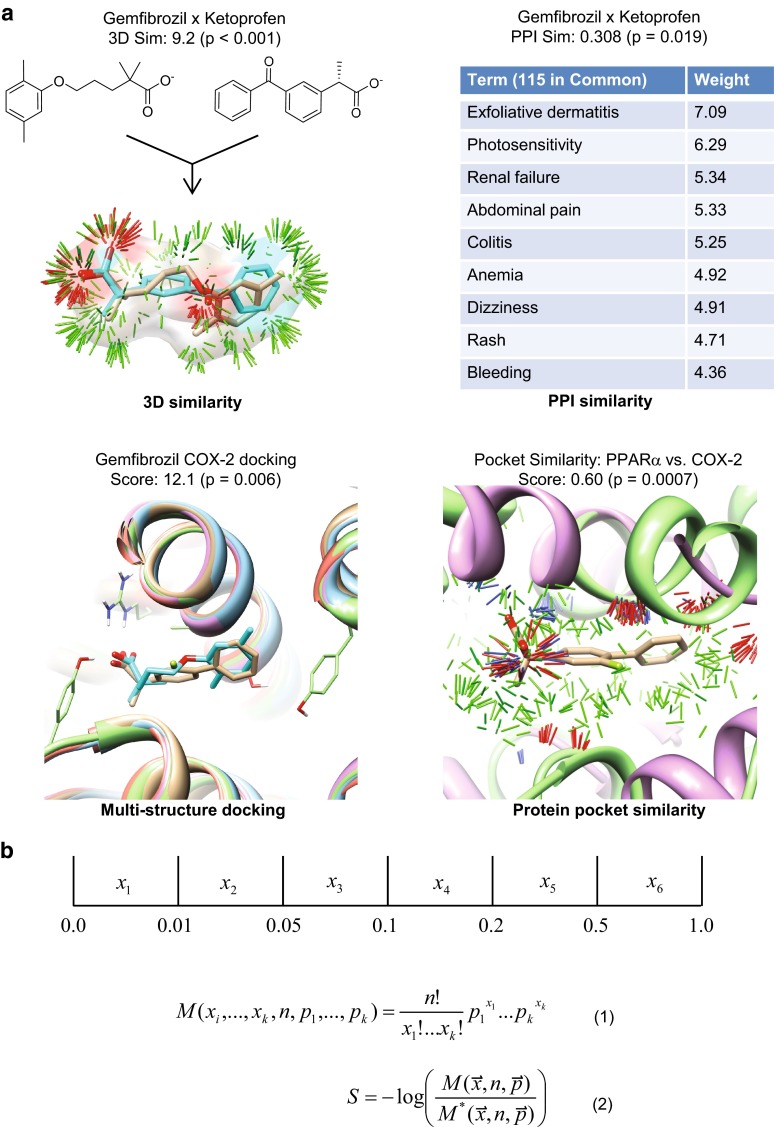
Combined computational approach for predicting ligand/target interactions. **a** Raw scores from four methodologies (3D chemical similarity, patient package insert text comparison, docking, and pocket similarity) are converted to *p* values which are then combined to yield a single log-odds score. For 3D and PPI similarities, the pairwise similarities are determined for a test molecule against a set of ligands that share a target. One representative pair for each method is shown. For docking, the test molecule is docked into multiple structures of the target in question. For protein similarity, the surfaces of 2 target protein pockets are compared. **b** Computation of log-odds score (S). We compute the likelihood that the observed set of *p* values is extreme using the multinomial distribution. The collective *p* values are binned (*top*), and the bin counts are computed. M is the likelihood of having observed such a set of *p* values, and M* is the same computation using the converse probabilities. The Log Odds score S combines the two. Positive S indicates that it is more likely that the molecule in question shares an activity with the ligand set than it does not

Given a particular query ligand (e.g. gemfibrozil, a PPAR*α* ligand), a prediction that it interacts with a new target (the COX enzymes) is based on three types of information: (1) chemical structures of ligands of the new target, (2) textual patient package insert (PPI) information for the query ligand and drugs that modulate the new target, and (3) multiple crystallographic structures of the known target and of the putative new target. Figure 1 summarizes the computational approach, which combines four methods, each of which measures molecular similarity or molecular complementarity. Panel (a) depicts the methods, which produce a *set* of scores (ligand structural similarity and PPI similarity) or a single score (docking and protein pocket similarity). Each score is transformed into a *p* value by making use of an empirically computed background score distribution [8]. Panel (b) illustrates how the resulting set of *p* values are combined to produce a single overall log-odds score.

Known drugs are the subject of investigation using the framework we describe in this work. This is because drugs have the richest annotation information available, including both phenotypic and structural information. However, we envision that the most important application of this framework is in the pre-clinical evaluation of candidate molecules. Clearly, the structure-based computations shown in Fig. 1 can be made for many putative off-targets, but the framework offers the ability to make use of a wide variety of phenotypic information, such as the increasingly common use of multi-target generalized assay panels. Similarity computations between vectors of biological assay data have been shown to be related to both structural similarity and to target preference [15], and scores arising from such comparisons can be incorporated into the log-odds framework. Linkages between a candidate molecule and an undesirable target or phenotype may suggest experimentally testable hypotheses that could avoid undesirable off-target effects.

Here, we show how multiple computational methods explain the mechanistic basis for the relationship between PPAR*α* and the COX enzymes. We present the first direct experimental evidence that fibrate drugs whose anti-lipemic effects are mediated through PPAR*α* are also COX inhibitors in vitro. This new finding suggests that the known anti-inflammatory effects of fibrates are mediated, at least in part, through direct inhibition of COX enzymes in vivo. Taken together, the results demonstrate the utility of a combined computational approach in identifying and understanding unexpected interactions between drugs and biological targets.

## Results

We have shown that the combination of molecular structural similarity combined with similarity computed from drug package inserts provides improved detection of true ligand-target interactions over use of single-mode similarity computations when controlling for false detection rates [9]. The study focused on 602 drugs and 91 diverse biological targets, with the emphasis being on computational validation of combining multiple ligand similarity methods in a blind prediction test on the ChEMBL database. Here, the focus is on including protein structural information and prospective validation of predicted interactions.

### Data mining putative drug/target relationships

The matrix of 602 drugs crossed against 91 biological targets from our previous study contained only a small fraction of cells with previously identified *bona fide* interactions. For this work, the remaining potential drug/target interactions were scored using the combination of 3D and PPI similarity (the most synergistic pair of similarity computations). To avoid focusing on unsurprising predictions such as the now well-documented target crosstalk between the ligands of amine-type GPCRs, and to increase the chances that protein structural information would be available, we filtered our results to include only those where the predicted target was an enzyme. Table 1 shows the ten top-ranked predictions, with log-odds scores ranging from 21.1–11.6.

**Table 1:** Top 10 log-odds predictions for enzyme targets

Drug	Known target	Predicted target	3D+PPI log-odds	Comment on prediction
Entecavir	HBV pol/RT	HIV-RT	21.1	Confirmed [10]
Telbivudine	HBV pol/RT	HIV-RT	20.7	No activity at clinical conc. [11]
Ribavirin	HCV RNA pol	HIV-RT	16.8	Confirmed [12]
Gemcitabine	RNR	HIV-RT	15.2	Supported [13]
Clofarabine	DNA pol	HIV-RT	14.4	Not tested
Valacyclovir	HSV DNA pol	HIV-RT	13.3	Confirmed [14]
Ganciclovir	HSV DNA pol	HIV-RT	13.1	Not tested
Levetiracetam	SV2	HIV-RT	12.9	Not tested
Gemfibrozil	PPAR*α*	COX-1	11.6	Shown in this work
Gemfibrozil	PPAR*α*	COX-2	11.6	Shown in this work

The top 8 results all had HIV reverse transcriptase (HIV-RT) as the predicted target. The top 7 predictions were for nucleotide analog drugs, most of which have viral or human polymerases as the intended target (but the target of gemcitabine is ribonucleotide reductase). A review of the literature showed that four of the HIV-RT activities were known (entecavir, ribavirin, gemcitabine, valacyclovir), three had not been tested or published (clofarabine, ganciclovir, levetiracetam), and only one (telbivudine) has been shown not to have anti-HIV-RT activity in vitro at clinical concentrations. The confirmatory literature on the nucleotide analog predictions was encouraging, but the predictions themselves were not terribly surprising and have not been experimentally tested in this work.

**Fig. 2:**
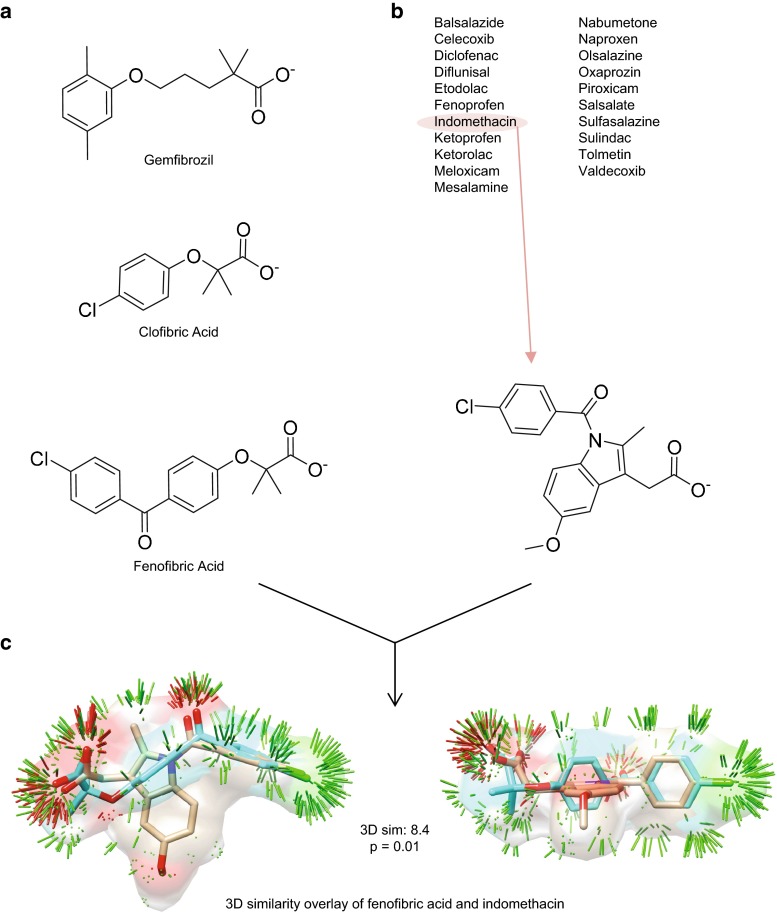
PPAR*α* and COX ligands. **a** 2D structures of three PPAR*α* agonists. **b** List of 21 NSAIDs annotated as COX inhibitors, with a representative 2D structure (indomethacin). **c** Optimal 3D superimposition of fenofibric acid (*cyan*) and indomethacin (*tan*). The *thin sticks* indicate regions of significant surface similarity, *green*-steric, *blue*-positive charge, *red*-negative charge. The raw similarity score of 8.4 corresponded to a *p* value of 0.01

However, the question of a linkage between PPAR*α* and the COX enzymes, for which multiple protein structures were available, offered a surprising prediction and an opportunity to explore the value of additional information. Gemfibrozil was present in our database as a free acid, as depicted in Fig. 2. A scan of our database identified two other fibrates (clofibrate and fenofibrate), but their structures were both represented by ester prodrug formulations. In vivo, the ester prodrugs are rapidly and completely converted by esterases to clofibric and fenofibric acid, respectively [16, 17]. Consequently, all computations discussed hereafter were performed on the free acid forms of all three fibrates. Figure 2 shows structures of the acids and also illustrates the optimal 3D overlay between fenofibric acid (*cyan*) and indomethacin (*tan*), corresponding to a raw score of 8.4 (equivalent to a *p* value of 0.01). Clofibric acid and fenofibric acid yielded 3D log-odds against the COX enzymes of 7.5 and 3.9, respectively. The PPI log-odds for fenofibric acid against the COX enzymes was 5.9 (PPI similarity could not be computed for clofibric acid due to the lack of a machine-readable package insert). Using all available *ligand-based* information, the overall log-odds scores for gemfibrozil, clofibric acid, and fenofibric acid were: 11.6, 7.5, and 7.0, respectively. To put these numbers in perspective, a systematic blind prediction test [9] suggested that log-odds scores of greater than 5.0 yielded correct ligand to target linkages 40–50 % of the time, with an upper bound on the false positive prediction rate of roughly 1–3 %.

### Synergy between protein structural and ligand-based information

Given the availability of multiple experimentally determined protein structures for both PPAR*α* and the COX enzymes, we assessed how the inclusion of protein structural information quantitatively affected the strength of the predicted association.

#### Multi-structure docking

Molecular docking was performed using a standard multi-structure protocol against both COX-1 and COX-2. The protocol employs multiple conformations of a given binding pocket and has been shown to significantly improve docking performance [6, 18]. The COX proteins have two spatially-distinct binding pockets, the cyclooxygenase site and the peroxidase site, each performing a separate enzymatic reaction. The cyclooxygenase site converts arachidonic acid to prostaglandin G2 and is the relevant binding site here. This site includes a hydrophobic channel, with a nominal difference of one residue between the COX isozymes, but the COX-2 site is 20 % larger [19].

**Fig. 3:**
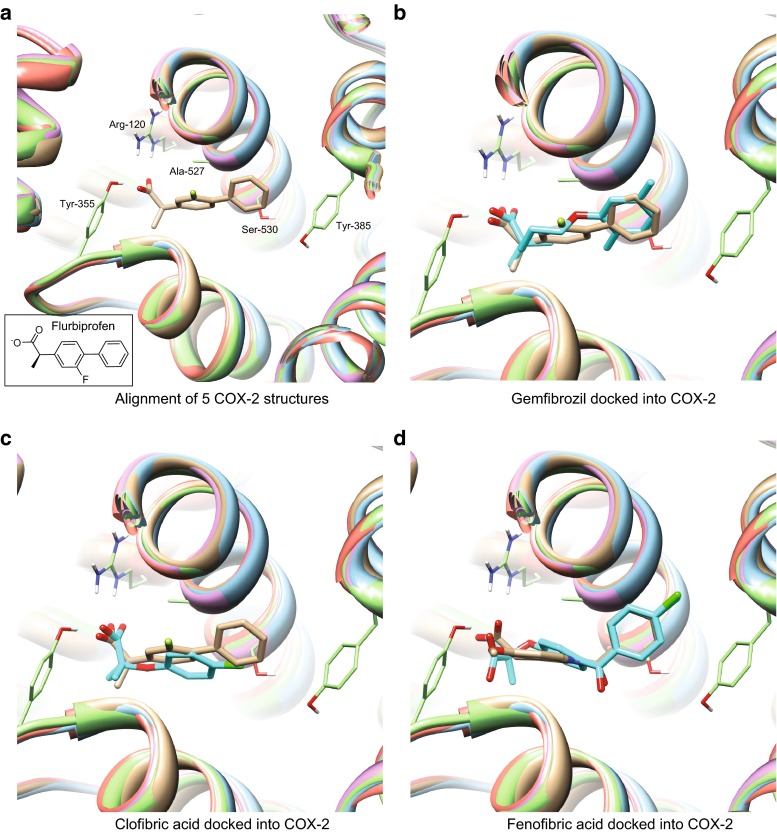
Multi-structure docking of PPAR*α* ligands into the cyclooxygenase site of COX-2. **a** Alignment of 5 COX-2 structures (*ribbon*) with key residues in *thin sticks* (*light green*). The cognate flurbiprofen ligand of 3RR3 is in *tan sticks* and the 2D structure is shown in the *lower left*. **b** Highest scoring docked pose of gemfibrozil (*cyan*) relative to native pose of flurbiprofen (*tan*) in 3RR3, *p* value = 6.0e−03. **c** Highest scoring docked pose of clofibric acid (*cyan*) relative to native pose of flurbiprofen (*tan*) in 3RR3. **d** Highest scoring docked pose of fenofibric acid (*cyan*) relative to the native pose of indomethacin (*tan*) in 4COX

The COX protein structures for docking included those with bound NSAIDs. For COX-1, these included PDB codes 2OYU, 3KK6, 3N8X, and 3N8Z (complexes with, respectively, an indomethacin analog, celecoxib, nimesulide, and flurbiprofen). The COX-2 structures were 1PXX, 3LN1, 3NT1, 3RR3, and 4COX, which are co-complexes with diclofenac, celecoxib, naproxen, flurbiprofen, and indomethacin. Protein pocket variants were mutually aligned using pocket similarity, as done previously [18]. The docking results were similar for COX-1 and COX-2, so representative COX-2 results are presented in Fig. 3. Panel (a) shows the 5 aligned COX-2 proteins (ribbons) with the cognate flurbiprofen ligand (tan sticks) of 3RR3. The labeled amino acids (light green, thin sticks) were previously identified as interacting with flurbiprofen in the co-crystal structure [20]. Specifically, the carboxylate of the drug forms a salt bridge with Arg-120 and a hydrogen bond with Tyr-355. The distal aryl ring forms van der Waals contacts with Gly-526–Ala-527 and stacks against Tyr-385. Note that the COX-2 binding pocket is relatively rigid, with sidechains exhibiting little movement on binding small inhibitors.

Figure 3b shows the highest scoring docked pose of gemfibrozil (cyan) relative to the native pose of flurbiprofen (tan) in 3RR3. Of the three PPAR*α* ligands, gemfibrozil yielded the most significant scores, corresponding to *p* values ≤0.01 against both enzyme isoforms. The other two ligands exhibited similar behavior, both in a numerical sense and in terms of the specific interaction geometry mimicking that seen with native COX inhibitors. Panel (c) shows the highest scoring docked pose of clofibric acid (cyan) relative to the native pose of flurbiprofen (tan) in 3RR3. Panel (d) shows the highest scoring docked pose of fenofibric acid (cyan) relative to the native pose of indomethacin (tan) in 4COX. The relative alignments between the fibrates and NSAIDs that were derived from the docking computations mirrored those seen from the 3D molecular similarity computations (see Figs. 1, 2).

As is often the case in non-native ligand docking, the use of multiple conformational variants of the proteins was important, despite the apparent lack of variation in the pockets by eye. The top poses for gemfibrozil and clofibric acid came from the flurbiprofen co-crystal structures (3N8Z and 3RR3). In contrast, the top docking scores for fenofibric acid resulted from the indomethacin structures (2OYU and 4COX). Collectively, the docking experiments strongly supported the hypothesis that PPAR*α* agonists are COX ligands and suggested rational poses for the fibrates in the cyclooxygenase pocket.

#### Protein binding site similarity

Given the ligand similarity and docking results, one would expect that there is some degree of binding pocket similarity between PPAR*α* and the cyclooxygenase site of COX-1 and COX-2. These proteins share no sequence similarity, either in a global sense, or at the level of the binding sites in question. Sequence comparison using BLASTp of Q07869 (PPAR*α*) against Q05769 (COX-2) yielded just two short matches, with overall coverage of just 8 %, each with E-values suggesting no significant match (>0.5). Full sequence alignment, using Needleman–Wunsch, produced just 15 % sequence identity, a level not considered to indicate statistically significant sequence similarity (N–W global protein alignment computed at blast.ncbi.nlm.nih.gov). In cases such as this, structural similarity may still exist, and the PSIM method has been shown to have utility in cases where sequence-based approaches do not [7, 21]. PSIM is a local, surface-based method to compare protein binding sites, and it is analogous to the small molecule comparison method whose results are illustrated in Figs. 1 and 2. Note, however, that the two proteins do not share the same SCOP/CATH fold and have a template-modeling structural alignment score of less than 0.4 (using TM-align, a global comparison method) [22].

**Fig. 4:**
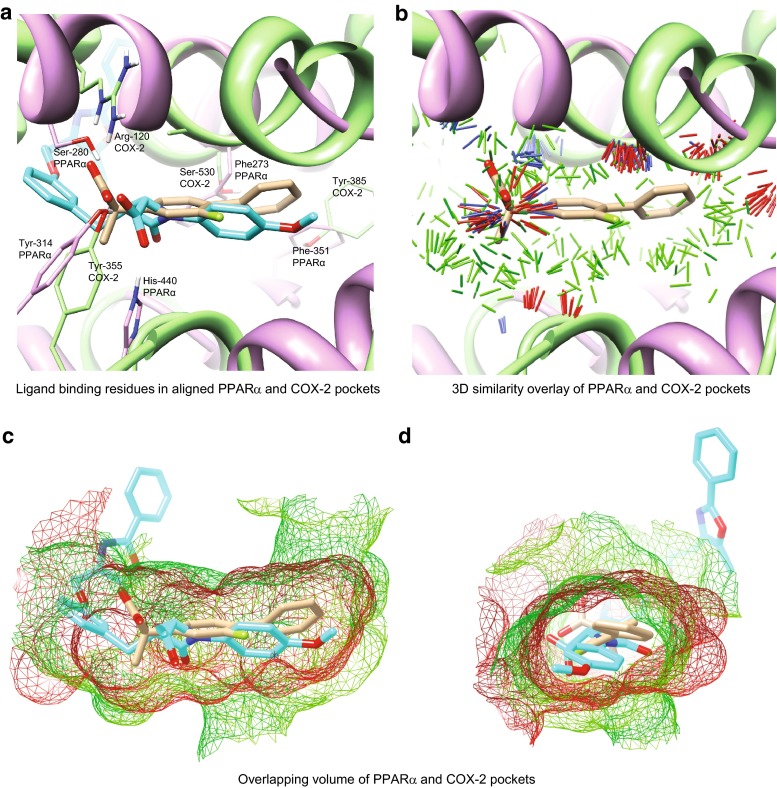
Binding site surface similarity between PPAR*α* and COX-2. **a** Alignment of PPAR*α* (2REW in *purple ribbon*) crystallized with BMS-631707 (*cyan*), and COX-2 (3RR3 in *green ribbon*) crystallized with flurbiprofen (*tan*). In *thin sticks* with labels are residues known to interact with bound ligands. PSIM score = 0.6, *p* value = 6.7e−04. **b** *Thin sticks* indicate regions of significant surface similarity, *green*-steric, *blue*-positive charge, *red*-negative charge. **c** In mesh overlay are the surfaces of the aligned proteins revealing the common volume of the binding pockets of COX-2 (*red*) and PPAR*α* (*green*). **d** 90° horizontal rotation of (**c**)

In order to compare the PPAR*α* and COX protein pockets, all 14 human PPAR*α* structures were obtained: 1I7G, 1K7L, 1KKQ, 2NPA, 2P54, 2REW, 2ZNN, 3ET1, 3FEI, 3G8I, 3KDT, 3KDU, 3SP6, 3VI8. The COX enzymes were represented by the nine structures used for docking (see above). An all-by-all similarity comparison of the 23 protein pockets was computed, and the PSIM raw similarity scores were converted to *p* values, as previously described [7]. Figure 4a shows the alignment of the highest scoring non-cognate protein pair, 2REW (PPAR*α* in purple ribbon) crystallized with BMS-631707 (cyan), and 3RR3 (COX-2 in green ribbon) crystallized with flurbiprofen (tan). The PSIM score was 6.0, corresponding to a *p* value of 6.7e−04. The maximal PSIM score for the COX isozymes was 9.0 (3N8Z and 3RR3) with a *p* value $\ll$0.001.

The labeled amino acids shown in thin sticks in Fig. 4a were identified as interacting with bound ligands and likely contribute to the similarity of the PPAR*α* and COX-2 pockets. Specifically, PPAR*α* Ser-280, Tyr-314, and His440 form hydrogen bonds with the carboxylic acid group of ligands, and Phe-273 and Phe-351 are known to line the hydrophobic cleft of the PPAR*α* pocket [23–26]. For flurbiprofen in COX-2, the carboxylate of the drug forms a salt bridge to Arg-120 and a hydrogen bond to Tyr-355, and the distal aryl ring of the ligand forms van der Waals contacts with Gly-526–Ala-527 and stacks against Tyr-385 [20]. COX-2 Ser-530 also interacts with the distal aryl ring of flurbiprofen and is the residue that is selectively acetylated by aspirin. Note the coincidence of ligand-binding residue pairs between the aligned pockets. For example, PPAR*α* Tyr-314 overlaps with COX-2 Tyr-355 and PPAR*α* Phe-351 overlaps with COX-2 Tyr-385. Figure 4b shows the 3D surface similarity of the pockets with thin sticks indicating regions of similarity, steric (green), blue (positive charge), and red (negative charge). There is a region of prominent polar similarity near the carboxylates of the bound ligands, and the green sticks indicate a significant common steric shape. The common pocket volume between PPAR*α* and COX-2 is highlighted in Fig. 4c, d where the protein surfaces are shown mesh (COX-2 in red and PPAR*α* in green). PPAR*α* and COX-2 clearly share quantitatively similar binding pockets, both in shape and surface charge, despite the lack of any primary sequence relationship between the two proteins.

Table 2 summarizes the number of *p* values and the log-odds predictions from the individual methods as well as the combination of the methods that relate gemfibrozil to the COX enzymes. Each individual method predicts the interaction, and the combined log-odds of 15.8 is much stronger than any single method alone. Beyond lending quantitative support to the hypothesized $\hbox {PPAR}\alpha /\hbox {COX}$ linkage, the results of the computations offer insight into the structural basis for ligand cross-talk, as illustrated in Figs. 3 and 4.

**Table 2:** Log-odds predictions for gemfibrozil interacting with COX enzymes

Method	Number of *p* values	Log-odds
3D Similarity	21	7.2
PPI similarity	21	8.7
Docking	2	3.4
Pocket similarity	2	3.4
Combined	46	15.8

### COX enzyme assays

In vitro assays were performed to directly test if gemfibrozil, clofibric acid, or fenofibric acid were COX inhibitors. The source of the enzymes for the assays were microsomal preparations from Sf9 cells transfected with recombinant human COX-1 or COX-2 [27], and the assay measured the conversion of the substrate arachidonic acid to PGE2. The steady state mean maximum plasma concentrations following the typical prescribed dose for the fibrates in this study are 240 μM for gemfibrozil [28], 1,000 μM for clofibric acid [29], and 50 μM for fenofibric acid [17]. Tests were performed at concentrations of 250 and 1,000 μM, based on the in vivo plasma concentrations. All three drugs exhibited inhibition of COX-1 at 250 μM: 48, 18, and 14 % for, respectively, fenofibric acid, gemfibrozil, and clofibric acid. At the higher concentration, inhibition increased for fenofibric acid (57 %) against COX-1. For COX-2, fenofibric acid also showed dose-dependent inhibition (23 and 41 % at the two concentrations).

For clofibric acid, marginal inhibition was seen at the higher concentration for COX-1 and at both concentrations for COX-2 (5, 13, and 11 %, respectively). Gemfibrozil also showed only marginal COX-1 inhibition at the higher concentration (8 %), and it exhibited a paradoxical behavior against COX-2, nominally *increasing* enzyme activity at the 250 and 1,000 μM concentrations (by 41 and 66 %). Such mixed phenomena are common in in vitro COX assays. In a recent study testing the COX modulatory activity of bioflavinoids, some ligands had maximum effect at 250 μM with a decline in activity at higher concentrations [30]. In addition, several compounds stimulated COX activity, and some compounds had opposite effects on COX-1 versus COX-2.

**Fig. 5:**
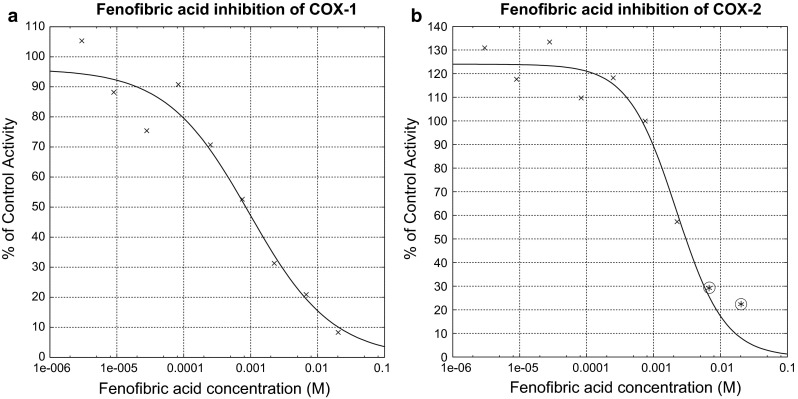
COX inhibitory activity of fenofibric acid. **a** Titration of COX-2 inhibitory activity, resulting in an IC50 of 950 μM. **b** Same plot for COX-2 inhibitory activity, resulting in a nominal IC50 of $\ge$ 2.2 mM. The IC50 was not determined definitively because assay detection was hampered at the two highest concentrations tested (*marked with circles*)

Because fenofibric acid showed the greatest COX inhibitory activity, full inhibitor titration assays were performed. Nine concentrations were tested, down to a concentration of 3 μM, using serial threefold dilution. The titration curves are shown in Fig. 5. The IC50 of fenofibric acid for COX-1 was 950 μM (Hill coefficient 0.7). This is comparable to that of NSAIDs such as acetaminophen and salicylic acid. Those drugs were shown to have IC50 values of $\sim 200$ and 500 μM, respectively, in a microsomal assay for COX-1 [31]. The effect of fenofibric acid on COX-2 was weaker by at least twofold, but still clearly dose-dependent, with a Hill coefficient of 1.2. For context in terms of selectivity, acetaminophen exhibits similar behavior, with recent data yielding IC50 values of 130 μM for COX-1 and 5,900 μM for COX-2 [32].

## Discussion

Our earlier work reported a probabilistic framework for relating ligands to putative off-targets, where the results of multiple types of ligand-based similarity computations were shown to have synergistic properties [8, 9]. Those studies made use of retrospective cross-validation and blind-testing approaches for methodological evaluation. Here, the focus has been on the prospective testing of predictions made using a generalization of the framework, which combines ligand similarity from both structure and text-based descriptions of clinical effects, docking computations, and protein pocket comparisons. Conceptually, the important contributions involve the exploitation of new information types that can be derived from protein structural information. Docking was used in an analogous fashion to ligand comparisons, with docking scores making a direct linkage to a putative target based on computations involving ligand fit into established active sites. The parallel ligand-based linkage makes comparisons between a ligand of interest to established small-molecule modulators of the putative target. Linkage of a different type, directly from one target to another, was made through the use of protein binding site comparisons. At present, we do not have an estimate for how often such surprising local structural similarities will exist between pairs of apparently unrelated proteins, but comprehensive computations on pairs of liganded protein binding sites are planned, in part to address this question.

The particular prospective result we describe is that $\hbox {PPAR}\alpha$ drugs such as fenofibric acid are also COX inhibitors at concentrations that are likely to be physiologically relevant in clinical application. Enzyme assay results showed that fibrates are indeed COX ligands in vitro and that fenofibric acid in particular has inhibitory COX activity similar to that of the less potent NSAIDs. The PPAR receptors are clinically important in anti-lipemic therapy, mediating one of the mechanisms by which fibrates lower high plasma triglycerides [33]. Fibrate use has more than doubled in the United States over the past decade, to nearly 1 % of the population [34]. Widespread and increasing fibrate use is driven by the heavy mortality burden of cardiovascular disease [35], underlining the need for understanding pharmacological crosstalk in this target and ligand category. $\hbox {PPAR}\alpha$ specifically upregulates the expression of genes for both the transporters and enzymes involved in the $\beta$-oxidation of fatty acids [36].

In addition to anti-lipemic benefits, PPARs and fibrates have been shown to have anti-inflammatory properties [37–39]. Fenofibrate was shown to reduce serum levels of tumor necrosis $\hbox {factor-}\alpha$ ($\hbox {TNF-}\alpha$) and $\hbox {interferon}-\gamma$ ($\hbox {IFN-}\gamma$) in hyperlipidemia patients [40]. Similarly, gemfibrozil was shown to increase survival in mice with an induced systemic inflammatory illness, likely through reducing excessive cytokine production [41]. In addition to reducing the expression of the inflammation mediators $\hbox {TNF-}\alpha$ and $\hbox {IFN-}\gamma$, $\hbox {PPAR}\alpha$ was demonstrated to negatively regulate COX-2 gene transcription [42]. Our results suggest that some of the anti-inflammatory effects of the fibrates may be due to *direct* interaction with the COX enzymes. That one of the effects of $\hbox {PPAR}\alpha$ activation is to downregulate COX-2 may point to a more general phenomenon. The binding sites of ligand-modulated transcription factors may have evolved to be sensitive to ligands of enzymes and receptors that are *downstream* of the transcription factors. This will be explored in future work by considering the similarity of ligand binding sites of transcription factors to those of the proteins they regulate.

A linkage between the COX enzymes and the PPARs had been noted by earlier investigators, stimulated by the observation that treatment of preadipocyte cell lines with indomethacin resulted in terminal differentiation to adipocytes [43]. Because PPAR$\gamma$ was known to be a regulator of adipocyte differentiation, indomethacin and other COX inhibitors were tested for PPAR modulatory activity. Indomethacin, fenoprofen, ibuprofen, and flufenamic acid were shown to be $\hbox {PPAR}\gamma$ and $\hbox {PPAR}\alpha$ agonists [44]. The biochemical contribution here is novel in that we have shown the converse: that established PPAR ligands have meaningful activity against the COX enzymes. Recently, dual-action anti-inflammatory small molecules have been sought to simultaneously inhibit the COX enzymes and activate the PPARs [45]. The structural relationships we have established may provide insight into ligand design.

**Fig. 6:**
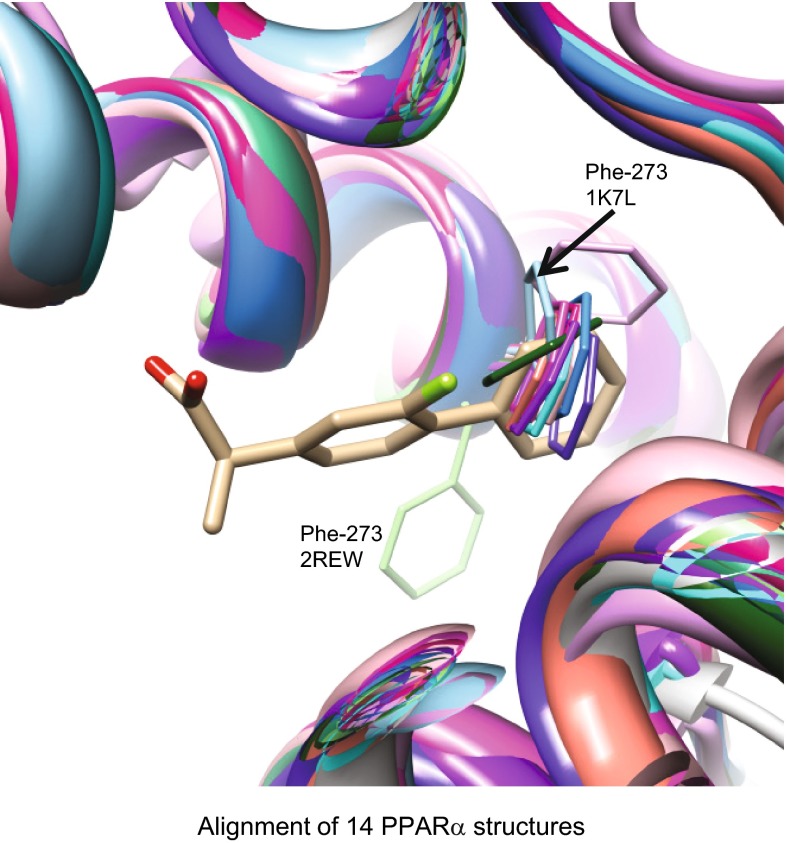
Shown is the alignment of 14 $\hbox {PPAR}\alpha$ structures (*ribbon*) with Phe-273 in *thin sticks*. Of the 14 structures, only 2REW has Phe-273 (*light green*) in an orientation that does not occupy the space of the distal aryl ring of the flurbiprofen ligand (*tan*) of the aligned 3RR3 COX-2 protein (not shown)

In this case, computations involving ligand similarity, docking, and protein pocket comparison each independently produced correspondences that all mutually agreed. Each produced a correspondence of parts, whether ligand to ligand, ligand to protein, or protein to protein. Given that the structural information included co-crystal structures for both targets, the cross-correspondences may be visualized (see Figs. 2, 3, 4) and seem to agree nearly atom for atom. For the ligand similarity and docking computations, the correspondence, while striking, is not surprising given that the molecules in question are relatively small organic acids. The protein alignment that gave rise to the significant pocket similarity score was more subtle, requiring the correspondence of an Arg/Tyr acid recognition element for COX-2 to a Ser/Tyr/His triad in $\hbox {PPAR}\alpha$. Further, the hydrophobic $\hbox {PPAR}\alpha$ pocket is only “open” in a single structure within the PDB, one which exhibits marked movement of a key residue in the binding site. Figure 6 shows the 14 aligned $\hbox {PPAR}\alpha$ proteins in ribbons with Phe-273 of each in thin sticks, with the ligand of the aligned 3RR3 COX-2 structure shown as well. Within the 2REW variant of $\hbox {PPAR}\alpha$, Phe-273 is rotated out of the space that is occupied by the aligned flurbiprofen. The structural importance of this residue has been highlighted with respect to ligand selectivity [23], conformational lability on ligand binding [46], and importance in direct ligand interactions [23, 24]. Several pairs of COX and $\hbox {PPAR}\alpha$ structures produced significant protein similarity scores, but the highest score arose from the particular case where Phe-273 was not occupying the ligand space of the aligned COX enzyme.

Each computational modality used here is subject to different biases and limitations, so the ability to combine diverse sources of information is a critical feature. Scores from any computations that relate a compound to another, a compound to a target, or a target to another target can be converted into probabilities. This only requires that each score has a monotonic interpretation (i.e. that a higher score suggests higher likelihood of linkage than a lower score, or vice versa).

Data associated with historical medicinal chemistry discovery projects may include assay data against particular targets, pre-clinical animal testing results (including textual descriptions of observed effects), or the results of broad standardized assay panels for pre-clinical evaluation. All such data should be amenable to the framework described here. We believe that hypotheses of off-target effects that are made based on such computations, when investigated experimentally, have the potential to reduce the frequency of discovering serious side effects during human trials.

## Methods

### Molecular data sets

The SPDB database of annotated drugs and targets has been described [8, 9]. Structures obtained from the PDB were downloaded from http://www.rcsb.org/pdb as Biological Assemblies.

### Computational methods

Surflex computational methods have been described in detail: 3D similarity [1, 47], PPI similarity and the log-odds computation including conversion of raw similarity scores to *p* values [8, 9], docking including multiple structures [6, 18, 48], and protein similarity including conversion of raw similarity scores to *p* values [7, 21]. All computations involving ligand similarity, docking, and protein pocket similarity were made according to standard protocols. Data, software, and computational protocols are available by request (see www.jainlab.org for details).

### Enzyme assays

COX assays were performed by Cerep Corporation (Redmond, WA). Briefly, human recombinant COX-1 and COX-2 were expressed in Sf9 cells and microsomes were prepared from the transfected cells as described [27]. Reactions proceeded for 5 min at room temperature with control or test compounds. The control inhibitor compounds were diclofenac for COX-1 and NS398 for COX-2. The assay measured the conversion of the substrate arachidonic acid to PGE2, and the detection method for PGE2 concentration was homogeneous-time-resolved-fluorescence [49].
